# Engineering genetically encoded FRET-based nanosensors for real time display of arsenic (As^3+^) dynamics in living cells

**DOI:** 10.1038/s41598-019-47682-8

**Published:** 2019-08-02

**Authors:** Neha Soleja, Ovais Manzoor, Parvez Khan, Mohd. Mohsin

**Affiliations:** 1 Department of Biosciences, Jamia Millia Islamia, New Delhi, 110025 India; 2 Centre for Interdisciplinary Research in Basic Science, Jamia Millia Islamia, New Delhi, 110025 India

**Keywords:** Biosensors, Fluorescence imaging

## Abstract

Arsenic poisoning has been a major concern that causes severe toxicological damages. Therefore, intricate and inclusive understanding of arsenic flux rates is required to ascertain the cellular concentration and establish the carcinogenetic mechanism of this toxicant at real time. The lack of sufficiently sensitive sensing systems has hampered research in this area. In this study, we constructed a fluorescent resonance energy transfer (FRET)-based nanosensor, named SenALiB (Sensor for Arsenic Linked Blackfoot disease) which contains a metalloregulatory arsenic-binding protein (ArsR) as the As^3+^ sensing element inserted between the FRET pair enhanced cyan fluorescent protein (ECFP) and Venus. SenALiB takes advantage of the ratiometic FRET readout which measures arsenic with high specificity and selectivity. SenALiB offers rapid detection response, is stable to pH changes and provides highly accurate, real-time optical readout in cell-based assays. SenALiB-676n with a binding constant (*K*_d_) of 0.676 × 10^−6^ M is the most efficient affinity mutant and can be a versatile tool for dynamic measurement of arsenic concentration in both prokaryotes and eukaryotes *in vivo* in a non-invasive manner.

## Introduction

All toxic metals in general and arsenic in particular is a potent carcinogen and an increasing threat to the ecological and global public health^1^. Arsenic (As), a ubiquitous, group V-A metalloid element with atomic number Z = 33 and atomic mass A = 74.9 naturally occurs throughout the earth’s crust and groundwater supplies^2,3^. High concentration of arsenic in the environment may result from natural geological processes such as weathering, erosion and volcanic eruptions or due to human activities like burning of fossil fuels, mining, industrial waste disposal, use of arsenical insecticides and ore smelting^2,4^. Arsenic can exist in both inorganic [arsenite As^3+^, arsenate As^5+^] and methylated organic [monomethylarsonic acid (MMA), dimethylarsinic acid (DMA) and trimethylarsine oxide (TMAO)] forms^1,2,5^. However, inorganic arsenical forms are potentially more toxic and may be associated with cancers of lung, skin, bladder, liver and kidney^6^. The immediate effects of acute arsenic poisoning include skin itching, vomiting, loss of appetite, abdominal pain, muscle cramps and diarrhoea. Systematic and chronic exposure may lead to serious health disorders like respiratory failure, skin ulcers, melanosis, keratosis, irritation of mucous membranes, cardiovascular diseases, developmental effects, vascular diseases (hypertension and Blackfoot disease) and cutaneous arsenicosis^1,2^.

A varied number of biological sensors have been described in the literature for analysis of bioavailable arsenic contaminants and are promising alternatives to classical methods for arsenic detection. Whole cell- and protein-based biosensors make use of reporters, such as green fluorescent protein (GFP)^4^, β-galactosidase^7^, luminescent (lux)^8^, phiYFP encoding a yellow florescent protein^9^ and organic fluorescent dyes acriflavine and Rhodamine B as fusion partners for transcriptionally active components^2^. Despite their potential application, such biosensors have not received much attention owing to their associated toxicity, limited reproducibility, high background signal and long incubation hours needed to achieve reliable results^5,6,9^.

Previous studies have discussed several mechanisms by which arsenic exerts its deleterious effects. Arsenic compounds have been known to alter protein-DNA, DNA-DNA interactions by inducing chromosomal aberrations, gene amplification, DNA hypomethylation, DNA damage and oxidative stress. They have also been shown to inhibit DNA repair, sister-chromatid exchanges by altering DNA methylation patterns and impair cellular respiration by inhibiting various mitochondrial enzymes. Enhanced cell proliferation, mitogenic stimulation and p53 suppression are the possible modes that contribute to the carcinogenic action of arsenic^1^. Therefore, it becomes very essential to understand the regulatory pathways/processes responsible for the uptake of arsenic and biochemical cellular events associated with it. Thus, there is an utmost need to come up with a simple, reliable, non-invasive, cost effective and highly sensitive method for determination of arsenic in trace amounts.

In the present study, we report a FRET-based nanosensor which is genetically encoded and measures the real time changes of this toxicant in a concentration-dependent manner inside the living cells. ArsR, the regulatory protein of *ars* operon is used as a recognition element along with two red-shifted variants of the GFP as reporter genes. In order to construct the nanosensor, ArsR protein was fused with ECFP and Venus, a yellow fluorescent protein (YFP) derivative at N- and C-terminus respectively. The sensor exploits the rate of energy transfer between the donor and acceptor fluorophore as an indicative measure for *in vitro* and *in vivo* analysis of arsenic at cellular level in both prokaryotes and eukaryotes.

## Results and Discussion

### Designing and construction of the FRET-based nanosensor

Arsenic (As) is adversely a toxic metallic element that negatively affects the human well-being and environment. Although arsenite As^3+^ is potentially more harmful than arsenate As^5+^, both the arsenical forms have been associated severe health issues such as chronic dermatitis and urothelial carcinoma^10^. Thus, there was a need to device a simple method that can monitor and measure the *in vivo* arsenic levels to get a better understanding of this metal ion transport, distribution and accumulation within the living cells. Transcriptional repressor ArsR of the *ars* operon has strong affinity for As^3+^ and its ability to bind arsenic ions makes it a promising candidate for developing FRET-based nanosensors.

**Figure 1:**
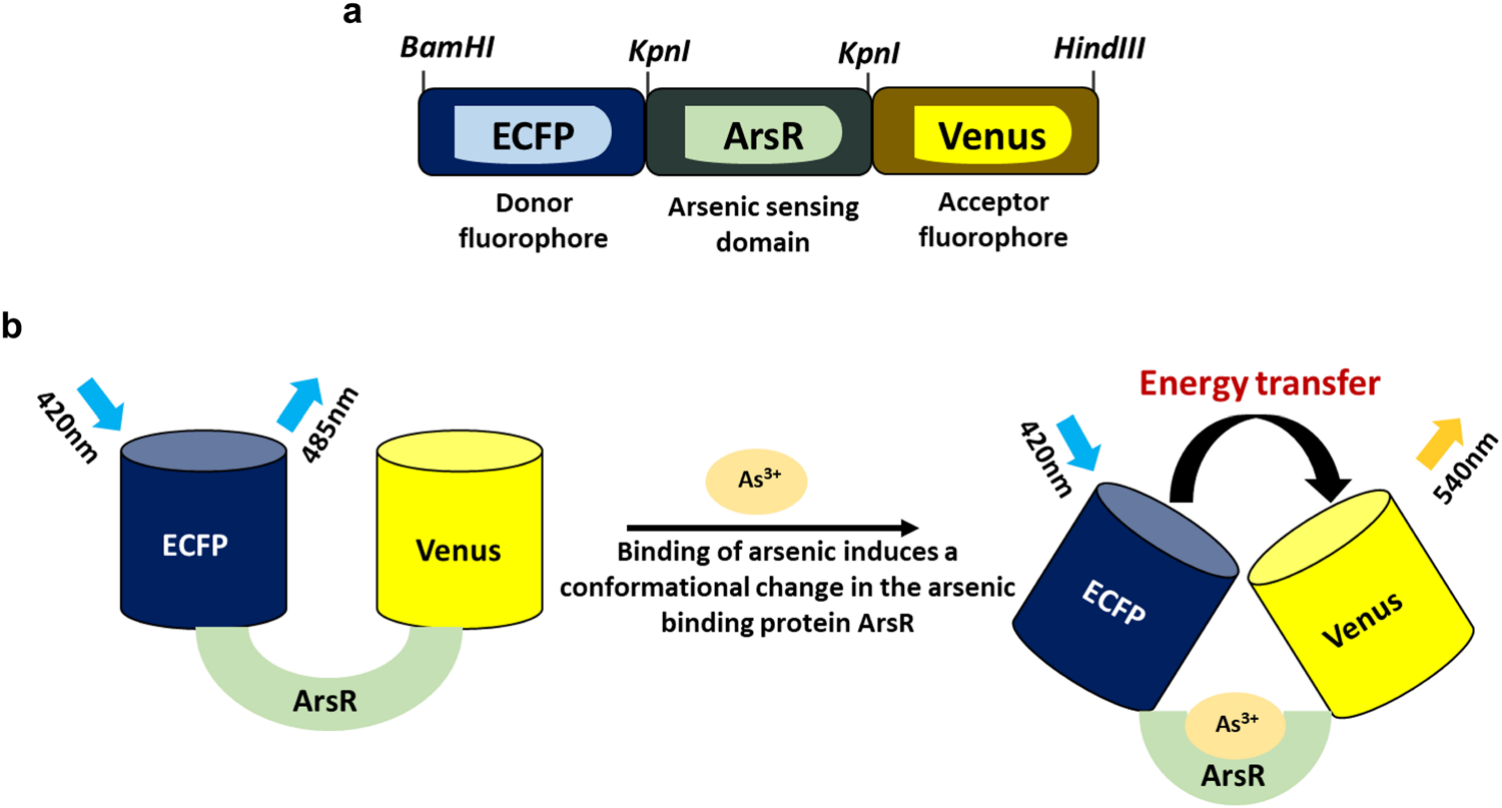
Illustrative representation of the nanosensor. (**a**) Construct map of SenALiB with position of the restriction sites. (**b**) General design of the developed sensor. As^3+^-induced conformational changes in the ArsR protein brings the two fluorophores ECFP and Venus in proximity, thereby transferring energy in the form of FRET.

Here, in this work, a genetically encoded sensor using fluorescent sensing technology has been developed for real time quantification of arsenic in a non-invasive manner. The fluorescent variants ECFP (donor) and Venus (acceptor) were linked with the arsenic responsive repressor ArsR at N- and C-terminus respectively to obtain a recombinant protein. The metalloregulatory protein ArsR of *E*. *coli* is a trans-acting repressor that senses arsenical forms present in the environemnt^3^. As a FRET pair, spectral variants ECFP and Venus of av-GFP (*Aequorea victoria*-Green fluorescent protein) were used for obtaining ratiometric changes in the flux rates of arsenic (As^3+^) at the cellular level. The nanosensor was successfully constructed using cloning strategies into a bacterial vector plasmid pRSET-B (Invitrogen, USA) generating an ECFP-ArsR-Venus sensor construct in pRSET-B. The pRSET-B vector offers a polyhistidine (6xHis) tag at the N-terminus for rapid purification of fusion proteins with nickel-chelating resin. The nanosensor construct was verified by sequencing, thereby, confirming its fidelity (Supplementary Fig. S1). A linear diagrammatic representation in Fig. 1a shows arrangement of the cleavage sites in the sensor construct. Figure 1b shows the representation of a FRET-based sensor in presence of arsenic. ECFP_ArsR_Venus construct map in bacterial, yeast and mammalian expression vectors are shown in supplementary Fig. S2. Fidelity of arsR gene was confirmed by sequencing (Supplementary Fig. S3). Simultaneously, the genes encoding for fluorescent proteins ECFP and Venus were successfully cloned in pRSET-B expression vector to generate constructs pRSET-B_ECFP, pRSET-B_Venus and pRSET-B_ECFP_Venus to carry out the fluorescence experiments and were used as controls throughout the study. pRSET-B_ECFP_Venus consisted of ECFP and Venus but lacking the sensing domain.

### Protein expression and purification

*E*. *coli* BL21-codon plus was transformed with the pRSET-B_ECFP_ArsR_Venus construct and expression of the protein was induced by adding 0.5 mM isopropyl β-D-1-thiogalactopyranoside (IPTG). With the aid of Nickel-nitrilotriacetic acid (Ni-NTA) His-tag affinity columns, recombinant protein was successfully purified. The fidelity of nanosensor protein was analysed and confirmed by running a 10% sodium dodecyl sulfate polyacrylamide gel electrophoresis (SDS-PAGE) and was named as SenALiB (Sensor for Arsenic Linked Blackfoot disease).

### Fluorescent emission scans of the nanosensor protein

The *ars* operon of *E*. *coli* plasmid R773 offers a well-characterized detoxification mechanism, thereby, conferring resistance to the arsenic, antimony and bismuth compounds^11^. The chromosomal operon contains an inducible repressor of As^3+^ (arsR), trans-acting metalloregulatory protein (arsD), ATPase (arsA), transmembrane As^3+^ transporter (arsB) and a reductase arsC which converts As^5+^ to As^3+^ ^12,13^. In absence of the metalloid As, ArsR binds as a homodimer to the *ars* promoter/operator blocking initiation of the ars transcription. However, even a low concentration of As^3+^ when present, binds to ArsR inducing a conformational change and thus, promotes the release of ArsR from the DNA (promoter/operator) for initiation of transcription of the *ars* operon^14^. SenALiB that contains ArsR as As^3+^ sensing domain was excited at λ_420_ nm and the spectral profile was monitored from 460–600 nm wavelength range in a monochromator microplate reader. The ArsR protein shows high affinity, sensitivity (can detect upto 10^−15^ M As^3+^) and selectivity towards arsenite^15^ and therefore has been exploited for developing SenALiB. Previously also, ArsR has been used as an As^3+^ sensing domain for the construction of whole-cell and microbial sensors^4–7,16^ but suffered large number of setbacks such as low/moderate sensitivity, heavy instrumentation, high cost, complex sample preparation and extreme toxicity.

**Figure 2:**
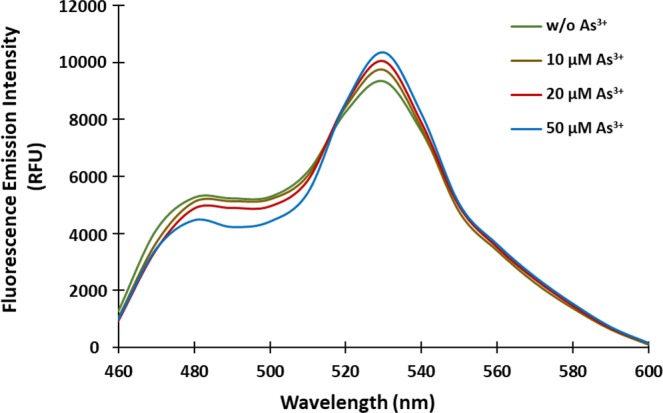
*In vitro* emission spectral scan of SenALiB. The spectrum was recorded in wavelength of range 460–600 nm. Addition of As^3+^ leads to a change in the relative fluorescence intensities of the donor and the acceptor fluorophore.

*In vitro* spectral analysis of the SenALiB shows corresponding changes in the fluorescence emission intensities of ECFP and Venus in presence of arsenite. In presence of As^3+^, the emission intensity of ECFP decreases and the intensity of Venus increases (Fig. 2). As^3+^-induced conformational changes in ArsR protein brings ECFP and Venus in proximity of 10 nm, thereby, transferring energy non-radiatively from donor to the acceptor molecule. This is consistent with the other genetically encoded sensors wherein upon metabolite binding, the two lobes of the PBPs twist and close in a “*Venus flytrap*” manner. This metabolite binding is translated into a FRET signal^17^. The result shows donor to acceptor energy transfer in the presence of As^3+^. FRET-based sensors are efficient tools for studying intracellular concentration of any metabolite^18^. Such sensors make use of metal binding proteins as recognition elements that undergoes ligand-dependent conformational dynamics to transfer energy between two FRET pairs in a non-radiative manner^19^. The efficiency (E) of energy transfer depends on the fluorophores’ distance from each other, dipole-dipole orientation and donor’s emission and acceptor’s absorption spectral overlap^20,21^. Earlier, organic fluorescent dyes have been used as FRET pair in fluorescent sensors^22^ but owing to their toxicity and difficult permeation into living cells, our approach is comparatively much more efficient.

### Stability analysis of the nanosensor protein

**Figure 3:**
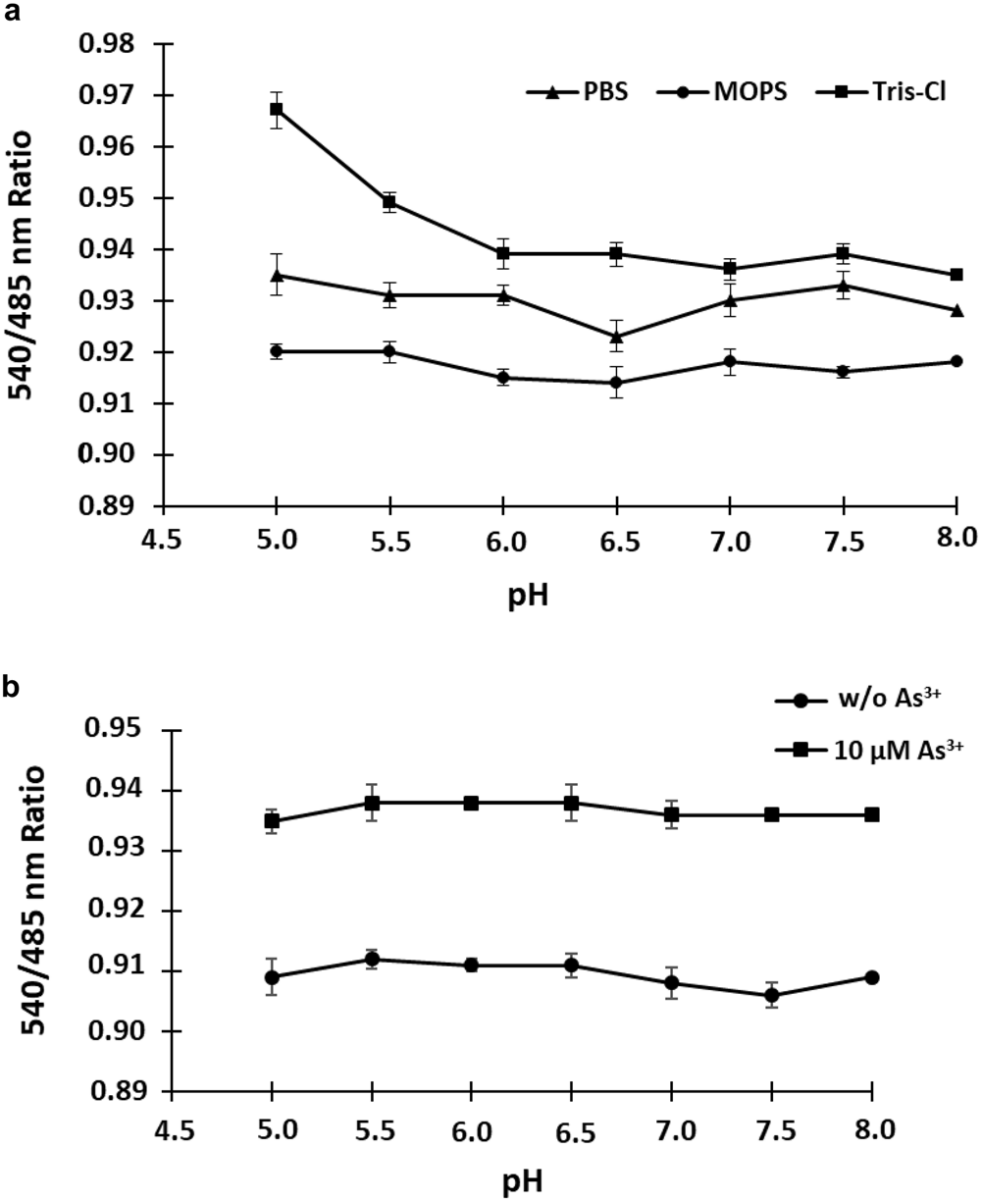
Buffer stability. (**a)** SenALiB was diluted in different buffers with altered pH range 5.0 to 8.0. Least change in intensity ratio was observed in case of 20 mM MOPS buffer. (**b)** Fluorescence emission intensity ratio change was recorded in 20 mM MOPS buffer in absence and with addition of 10 µM arsenic. Data points are expressed as mean ± SD from n = 3.

The sensor’s stability was determined in phosphate buffer saline (PBS), 3-(N-morpholino) propanesulfonic acid (MOPS) and Tris-Cl. The protein was characterized by FRET method in the pH range (5.0 to 8.0) of different buffers as change in the emission intensity (Em_540_/Em_485_). When tested for the stability, it was observed that SenALiB shows non-significant changes in the dual emission intensity ratio in 20 mM MOPS buffer (Fig. 3a). 20 mM MOPS buffer of physiological pH 7.2 was used for diluting the sensor protein in further experimental assays. The different spectral properties exhibited by the proteins ECFP and Venus make them an attractive and suitable FRET pair. Enhanced yellow fluorescent protein (EYFP) variant, Venus shows reduced environmental sensitivity, is brighter and has significantly lower pKa (~6.0) which makes this protein less pH sensitive and a desirable acceptor^23,24^. It has been previously established that a novel mutation, F46L in the SEYFP (super-EYFP)-F46L variant Venus accelerates the rate-limiting oxidation step of chromophore maturation at 37 °C leading to the fluorescence enhancement of YFP. Mutations, such as F64L/M153T/V163A/S175G in the rapidly-maturing variant Venus, makes it less sensitive to acidosis^25^ and halides (Cl^–^). Venus has been used as an acceptor molecule^26^ for developing lactate and pyruvate FRET sensors^27,28^.

FRET ratio was also recorded by diluting the sensor protein in 20 mM MOPS buffer of varying pH range (5.0 to 8.0) in absence as well as in the presence of 10 µM As^3+^ using a fluorescent monochromator microplate reader (Biotek, USA) with 96-well. In the acidic conditions, i.e., upto pH 7.0, the ratiometric change was quite significant but in the alkaline physiological range (7.0 to 8.0), the sensor protein seems to be comparatively stable as the change in pH in this range triggers minimal variation in the emission intensity ratio (Fig. 3b). Since physiological pH of *S*. *cerevisiae* is close to 7.0, the pH stability of SenALiB makes it an appropriate choice for studying the *in vivo* level of arsenic^29^.

### Fluorescence response

In order to rule out the effect of As^3+^ on individual fluorophore (ECFP/Venus), we made the respective constructs pRSET-B_ECFP, pRSET-B_Venus and pRSET-B_ECFP_Venus without the binding domain. The proteins were then expressed, purified and effect of As^3+^ on each fluorophore were studied using fluorescent spectroscopy. Further, in order to see the structural changes that might occur after As^3+^ interaction with each protein, we also used internal florescence of Trp as a probe. For this, the fluorescent proteins were excited at λ_280_ nm and the respective emission spectra were recorded within the wavelength range of 310–400 nm. It was found that, on increasing the concentrations of As^3+^ the internal fluorescence of ECFP/Venus was decreased but the quenching so observed was not much appreciable or significant (Supplementary Fig. S4). Furthermore, the proteins were excited at λ_420_ nm and the three emission spectra were recorded in 460–600 nm wavelength range. In this case too, added As^3+^ does not trigger much change in the fluorescence intensity (Supplementary Fig. S5). These observations clearly suggested that As^3+^ have very weak or no affinity towards these fluorophores. The observed changes in the fluorescence intensity of ECFP/Venus were might be due to the dilution effect.

### *In vitro* assays of the SenALiB

**Figure 4:**
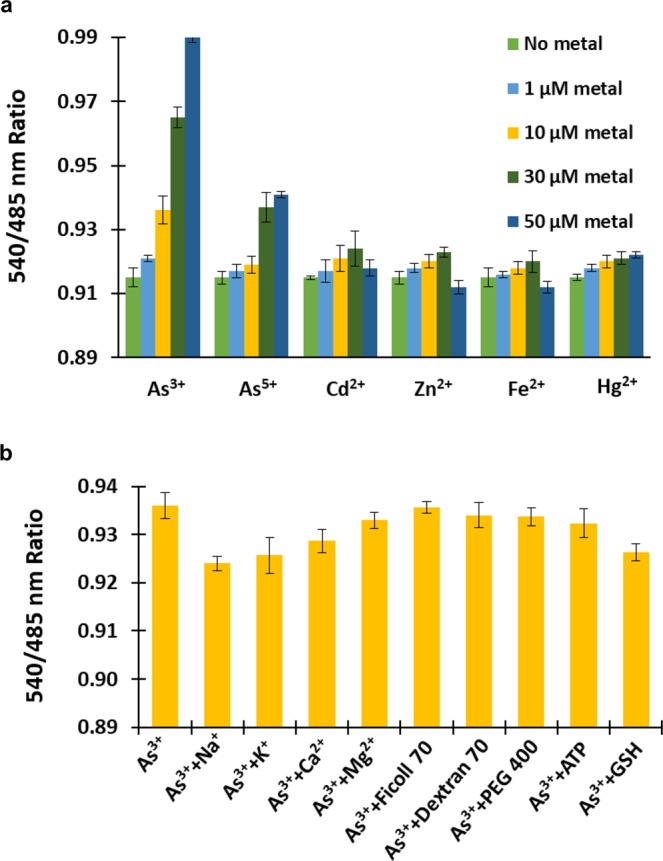
*In vitro* assay characterization. (**a**) Metal ion specificity of the purified protein was measured and maximum change in the ratio was obtained with As^3+^. (**b**) Change in the acceptor-to-donor fluorescence ratio on adding potential intracellular interferents. Data points are expressed as mean ± SD from n = 3.

The detection specificity of SenALiB was tested by measuring the change in the emission intensity (Em_540_/Em_485_) ratio using a microplate assay as shown in Fig. 4a. In addition to arsenite (As^3+^), metals arsenate (As^5+^), cadmium (Cd^2+^), zinc (Zn^2+^), iron (Fe^2+^) and mercury (Hg^2+^) were also taken for the study. Coordination of As^3+^ to ArsR causes significant enhancement of the dual emission intensity ratio, whereas other metal ions including As^5+^ induces slight variation in the ratio, indicating that the nanosensor is selective towards As^3+^. High specificity and selectivity of ArsR protein for As^3+^ is contributed to the fact that binding of As^3+^ to amino acid residues Cys-32 and Cys-34 of arsR produces a conformational change in the protein which then gets released from the promoter/operator region and transcription of the *ars* operon is initiated^6,30,31^. This is in accordance with our findings that the FRET ratio change was not that significant when As^5+^ was added to the sensor protein. As previously established, despite the fact that binding site on arsR protein cannot recognize As^5+^, bacterial cells when incubated with arsenate ion shows two-fold times decrease in the light output. This may be due to the difference in the rate of enzymatic reduction of As^5+^ to As^3+^ by glutaredoxin and the interaction of As^3+^ with the arsenite-binding site on ArsR protein^7^. Here, SenALiB focuses on the detection and quantification of arsenite (As^3+^). However, it can detect As^5+^ also but to a very less extent.

Furthermore, competition experiments with the potential intracellular interferents such as biologically essential metal ions (Na^+^, K^+^, Ca^2+^ and Mg^2+^), molecular crowders (Ficoll 70, Dextran 70 and polyethylglycol PEG 400), adenosine triphosphate (ATP) and glutathione (GSH) were also performed in presence of 10 µM As^3+^. Slight but non-significant ratiometric fluorescence changes were obtained with all the species (Fig. 4b). It was therefore, concluded that these species when co-exist do not interfere with the As^3+^ sensing. Such approach has been earlier used while developing genetically encoded fluorescent Mg^2+^, K^+^ and H_2_O_2_ indicators^32–34^. Synthetic polymers (e.g., Ficoll 70, Dextran 70 and PEG 400) were used as “crowding agents” and their effect on the nanosensor was analysed to mimic the crowded conditions of the living cells. Proteins are usually tested for their efficacy in presence of crowders to study their stability and protein-protein interactions^35^.

**Figure 5:**
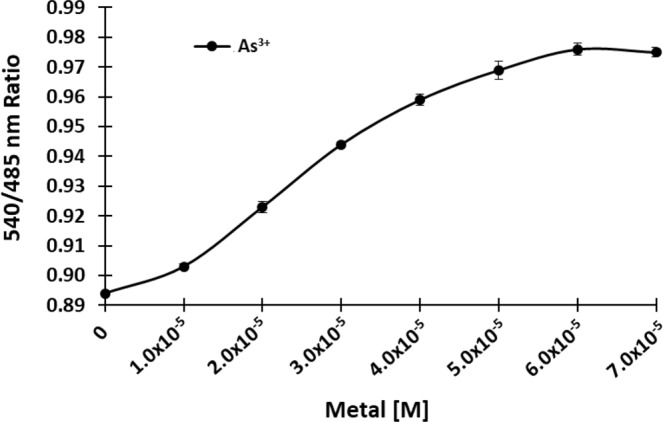
Ligand binding isotherm of SenALiB. FRET ratio change recorded in the presence of different As^3+^ concentrations to get a saturation curve. Data points are expressed as mean ± SD from n = 3.

Level of arsenic toxicity mainly depends on its chemical forms. The trivalent arsenical form As^3+^ has strong affinity for thiol or the sulfhydryl groups of proteins as it reacts with the cysteine residues of proteins via trigonal pyramidal geometry and can inactivate up to 200 enzymes. These interactions alter the conformation and structure of specific proteins, thereby effecting vital organ systems. Alternatively, As^5+^ acts as a phosphate analogue, is comparatively less toxic and competes with the phosphate ion transporters in several metabolic pathways disrupting various cellular processes^3,36,37^. Affinity of the purified SenALiB was analysed by incubating the sensor protein with different concentrations of arsenite (As^3+^) and measuring the 540/485 nm ratio. Fluorescence analyses showed that with the addition of As^3+^ in the range of 1 µM to 70 µM, there was a concentration dependent increase in FRET ratio and saturating at 60 µM following a sigmoidal curve. The purified wild type (WT) sensor binds As^3+^ with 25.97 µM *K*_d_ value, showing a prominent change of 0.082 in the FRET ratio (Fig. 5). Thus, SenALiB can serve as a promising sensing tool to monitor and measure the *in vivo* As^3+^ levels to get a better understanding of this metal ion transport, distribution and accumulation within the living cells.

### Affinity variants by site-directed mutagenesis

**Figure 6:**
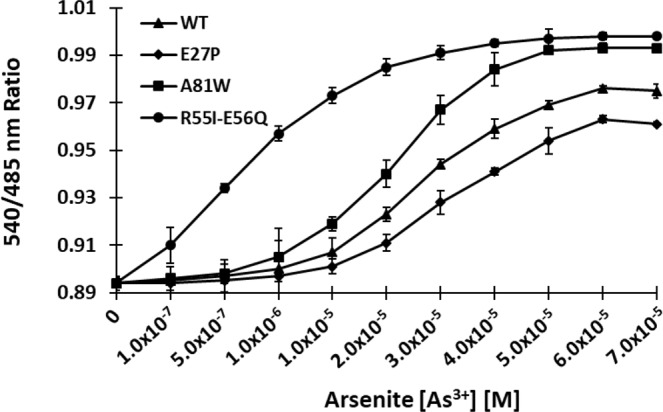
Ligand-dependent FRET ratio change for WT sensor and affinity mutants. The affinity mutants E27P and A81W were created and compared with the WT sensor. Data points are expressed as mean ± SD from n = 3.

A set of mutants were generated to augment the dynamic range of arsenic detection. The physiological range of detection was widened by developing mutant sensors after introducing point mutations in the ArsR protein of the construct ECFP-ArsR-Venus. Point mutations were introduced in the amino acid residues yielding the affinity sensors. Mutations were confirmed by sequence analysis (Supplementary Fig. S6). The affinity mutants together with the WT sensor covered the range of 0.20 µM to 58.96 µM arsenic detection (Fig. 6). The calculated *K*_d_ of WT (SenALiB-25µ), E27P (SenALiB-35µ), A81W (SenALiB-14µ) and the double mutant R55I-E56Q (SenALiB-676n) were 25.97 µM, 35.97 µM, 14.48 µM and 0.676 µM respectively (Table 1). The affinity variants can be used as controls for excluding artifacts^14^. SenALiB-676n was found to be the most efficient nanosensor created and was further used to carry out *in vivo* measurement of As^3+^ flux in eukaryotes at real time. The affinity mutants created enhanced the sensor’s detection range and can measure the intracellular arsenic levels physiologically at different scales. Previously, intensity-based approach measuring FRET ratio between the donor and acceptor molecules^38^ have been implemented in developing sensors for metabolites such as glucose, glutamate, thiamine and lysine^39–42^.

**Table 1:** Binding properties of SenALiB-WT and the sensor variants.

Name of nanosensor^a^	Mutation	*K*_d_ value (M)	Range of detection^b^ (µM)
SenALiB-25µ	WT	25.97 × 10^−6^	5.00–53.78
SenALiB-35µ	E27P	35.97 × 10^−6^	2.00–45.50
SenALiB-14µ SenALiB-676n	A81W R55I-E56Q	14.48 × 10^−6^ 0.676 × 10^−6^	6.00–58.96 0.20–55.67

Binding constants (*K*_d_) determined *in vitro*.

^a^Number alongside the sensor name stands for the *K*_d._

^b^Effective quantification range is the range of detection between 10% and 90% saturation of the nanosensor.

### *In vivo* characterization of SenALiB-676n in *E*. *coli*

**Figure 7:**
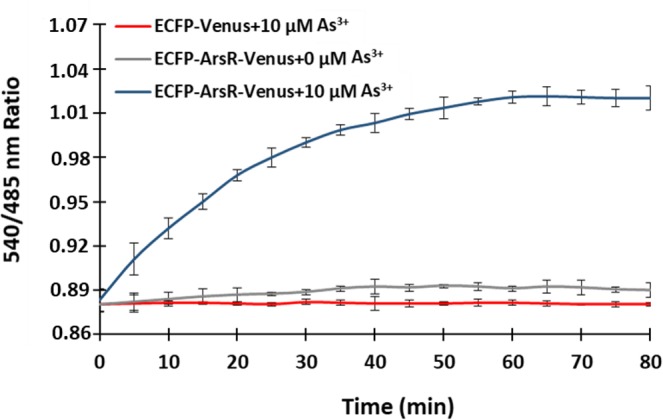
*In vivo* analysis of SenALiB-676n. Bacterial cell suspension expressing the nanosensor was incubated in absence and with addition of 10 µM As^3+^ and the FRET ratio change was recorded for 80 min. As a negative control, 10 µM As^3+^ was added to the ECFP-Venus system and the ratiometric change was obtained for the defined period. Data points are expressed as mean ± SD from n = 3.

SenALiB-676n was expressed in the bacterial cells to monitor the changes in the 540/485 nm ratio in *in vivo* with the addition of As^3+^ externally. The ratiometric change was recorded for 80 min in totality with an interval gap of 5 min. The Venus/ECFP ratio of the bacterial cell suspensions expressing SenALiB-676n showed a sharp increase with 10 µM As^3+^ initially and seemingly got saturated at 60 min indicating accumulation of arsenic in the bacterial cells (Fig. 7). An increase in the FRET ratio indicates an increase in the cellular arsenic levels as a result of combined uptake and transport^43^. This is in accordance with the As^3+^ uptake by *E*. *coli*^15^ and previously published reports that the ratiometric increase is indeed because of the uptake and transport mechanism of the metabolites^43,44^. Negative control pRSET-B_ ECFP_Venus was then expressed in *E*. *coli* as stated earlier and FRET pair acceptor-to-donor fluorescence ratio was calculated. It was observed that upon adding As^3+^ emission intensity ratio (Em_540_/Em_485_) change was negligible, clearly indicating that energy transfer is not occurring when the As^3+^ sensing domain ArsR is absent from the construct. Proving the idea that conformational alteration in the binding domain of the construct leads to FRET. High spatio-temporal resolution can be achieved by using such FRET sensors that provides detailed knowledge of the flux rates and intracellular concentrations of the metabolites^43,45^. The *in vivo* response curve was obtained by adding arsenic at concentration 10 µM to the cell suspension in 96-well microtiter plates. Confocal images indicated that the sensor protein has been successfully expressed in the bacterial cells (Supplementary Fig. S7).

### Monitoring of arsenic flux rates in yeast

**Figure 8:**
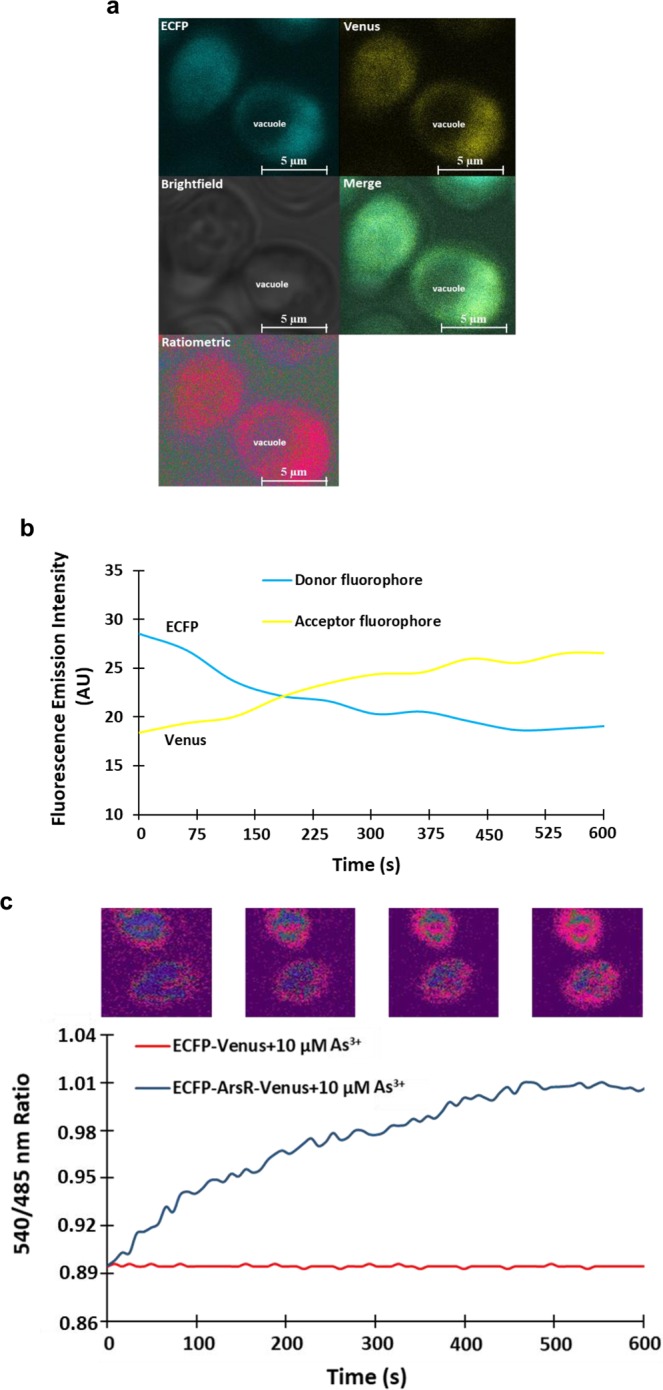
*In vivo* quantification of the nanosensor. (**a**) Confocal image of SenALiB-676n expressed in the cytosol of *S*. *cerevisiae*. (**b**) Time-course emission intensities analysis of the donor and acceptor fluorophores on addition of As^3+^. (**c**) The graph indicates FRET ratio change in the cytosol of a single yeast cell and was compared with that of control after addition of 10 µM As^3+^ along with ratiometric images.

So far, no fluorescent sensor has monitored the changes in the level of As^3+^ concentration in the eukaryotic system in a non-invasive manner. To test the efficacy of SenALiB-676n in yeast, it was transferred using gateway cloning in the vector pYEST-DEST52 (Invitrogen, USA), and further transformed into *Saccharomyces cerevisiae* (*S*. *cerevisiae*) for expression. ECFP-Venus construct was also prepared in pYEST-DEST52 to be used as a negative control. Liquid yeast extract peptone dextrose (YEPD) media was used to grow the yeast cells that were then induced in presence of 3% galactose to express SenALiB-676n. Ratiometric imaging was carried out using Leica confocal microscope equipped with LAS-AF software (Leica, Wetzlar; excitation 420/20 nm; emission filters 485/20 nm and 540/20 nm for ECFP and Venus respectively). Confocal images of yeast cells showed an unstained vacuole and a highly fluorescent cytosol indicating the expression of SenALiB-676n in the cytosol (Fig. 8a). Change in the arsenic concentration was visualized in the cytosol of a yeast cell with the addition of As^3+^ in terms of changes in the fluorescence intensities of the two fluorophores (Fig. 8b). With the addition of 10 µM As^3+^, a change in the FRET ratio is obtained indicating the uptake of arsenic in the cytosol, where it gets recognized by SenALiB-676n. Venus/ECFP emission intensity ratio increased considerably from 0.894 at 0 sec to 1.005 at 7 min which then reaches at saturation level while no change in the ratio was acquired with the ECFP-Venus only (Fig. 8c). Showing that the FRET is the result of conformational changes in ArsR upon binding of As^3+^. Similar FRET based approach has been used earlier to study the uptake of metabolites by measuring the dynamic changes in the FRET ratio^45^.

### Real time monitoring of SenALiB-676n in HEK-293T cell line

**Figure 9:**
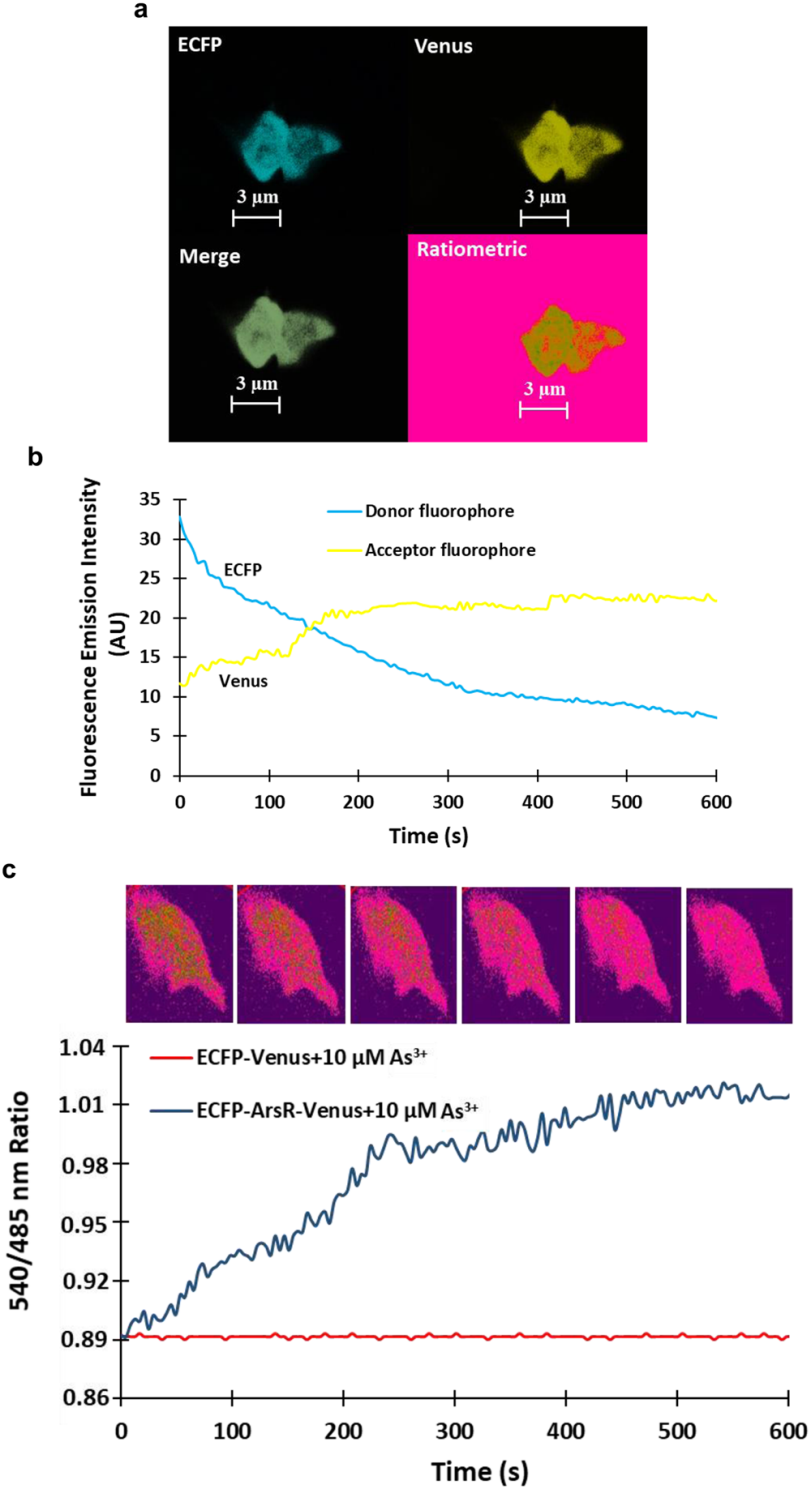
Real-time ratiometric analysis of SenALiB-676n in mammalian cell line. (**a**) Confocal imaging of the nanosensor expressed in the HEK-293T cell line. (**b**) Change in the dual emission intensities of the donor and the acceptor molecules with time. (**c**) Venus/ECFP ratio change in the presence of 10 µM As^3+^ in a single HEK-293T cell for 10 min. The graph shows the ratiometric change with respect to the negative control for the defined period.

SenALiB-676n sequences were transferred to pcDNA3.1 (−) vector (Invitrogen, USA) to express the variant in mammalian cell. Transfection of SenALiB-676n in mammalian human embryonic kidney (HEK)-293T cells was performed to analyse the *in vivo* activity of the sensor in real time. Images of HEK-293T cells expressing the sensor were obtained using confocal microscopy (excitation 420/20 nm; emission lasers 485/20 nm and 540/20 nm). Successful expression of SenALiB-676n was observed in the mammalian cell line as depicted in the *in vivo* ratiometric images (Fig. 9a). By the addition of As^3+^, emission intensity of ECFP decreased with time with a concurrent increase in the Venus emission intensity (Fig. 9b). FRET ratio was recorded and found to be increased with addition of 10 µM arsenic in a time-dependent manner. The 540/485 nm ratio at the basal level was 0.896 at 0 sec and increased rapidly reaching to a saturation level of 1.010 after 7 min of incubation in presence of As^3+^. HEK-293T cell line transfected by pcDNA3.1 (−) containing the ECFP-Venus construct as the negative control, no significant changes in Venus/ECFP ratio were obtained (Fig. 9c). This shows that SenALiB-676n is responding *in vivo* to As^3+^ that has been added externally by showing As^3+^-dependent increase in the dual emission intensity ratio. Mammalian expression of similar genetically encoded FRET-based sensors have proved that these sensors allow localization and detection of a metabolite within the cellular and sub-cellular compartments in a non-invasive manner, as demonstrated in case of cadmium and lysine sensors^42,46^.

### Cytotoxicity studies

**Figure 10:**
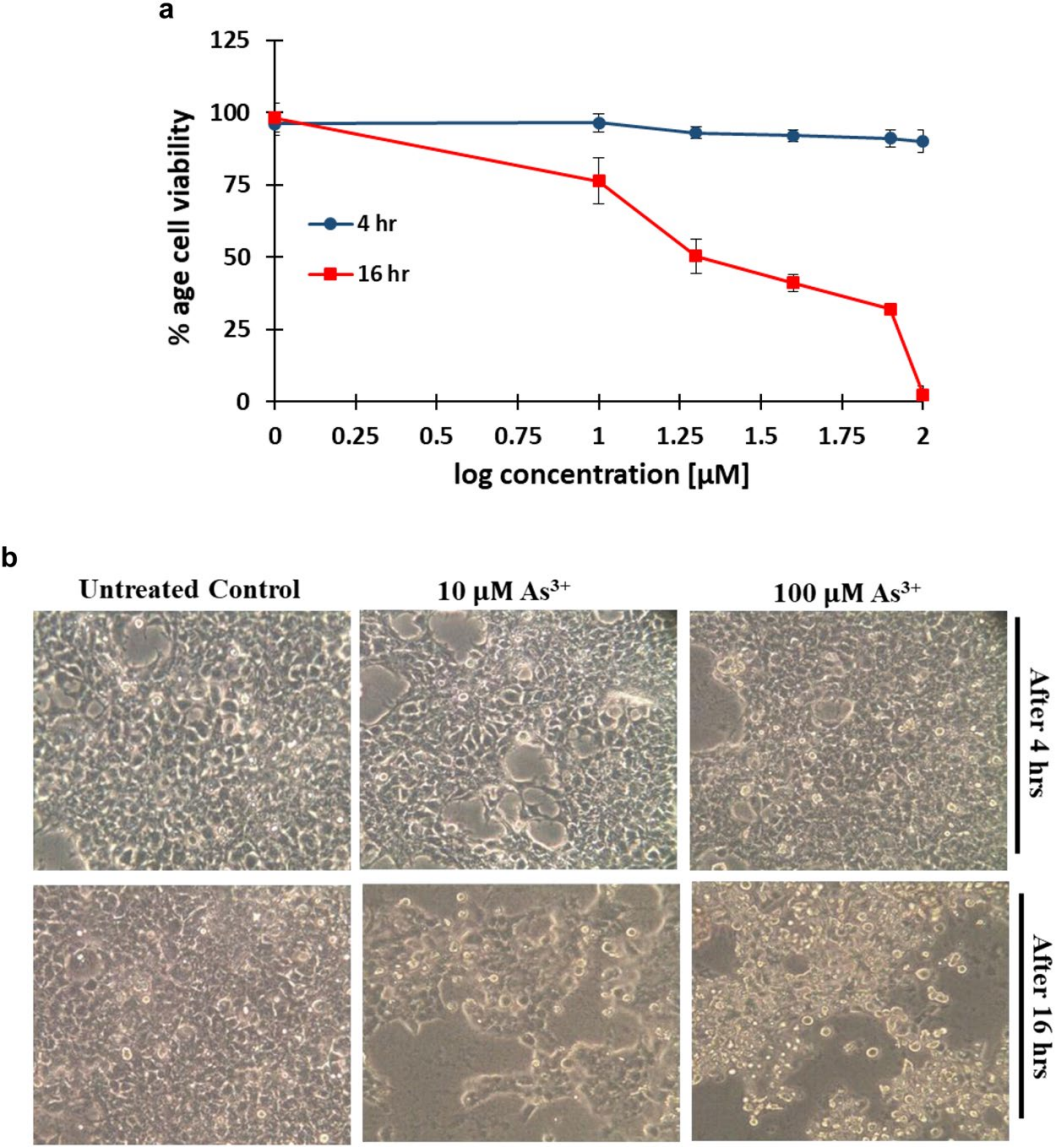
Cytotoxicity studies. (**a**) Cell viability of HEK-293T cells, evaluated through MTT assay. Cells were treated with increasing As^3+^ (0–100 μM) concentrations for 4 and 16 hrs. Percent cell viabilities were estimated in regard to the untreated control cells. Data points are expressed as mean ± SD from n = 3. (**b**) Representative images (20 X magnification) showing morphology of HEK-293T cells under different treatments of arsenic 10 and 100 µM taken on phase contrast inverted microscope.

Arsenic exposures are toxic for the human cells^47,48^, so in order to observe the toxic potential of As^3+^ on HEK-293T cells, 3-[4,5-dimethylthiazol-2-yl]-2,5-diphenyltetrazolium bromide (MTT) assay was performed. As cells were transiently transfected for measuring the FRET ratio and treatments were given at the time of measurements. Thus, cell toxicity studies were performed for short time (4 hr) and longer time (16 hr). Cell viability results showed that the treatment of As^3+^ is non-toxic upto 4 hr but induces significantly toxicity after 16 hrs exposure (Fig. 10a). We also examine the morphological changes in HEK-293T cells after 10 µM and 100 µM As^3+^ treatment using phase contrast inverted microscope. It was found that arsenic did not affect the morphology of HEK-293T cells when treatment was given for 4 hrs, while after 16 hrs treatment the morphology of cells altered drastically (Fig. 10b). Results are clearly suggesting that short time of exposure of As^3+^ did not affects the morphology neither induces toxicity to HEK-293T cells but it becomes toxic when the treatment time extends.

## Conclusions

Taken together, a genetically encoded fluorescent nanosensor was developed that can quantify and assess the cellular As^3+^ levels. *In vitro* and *in vivo* experiments showed that SenALiB is responding to the changing level of As^3+^ specifically. Creation of variants by mutation changes the physiological range of detection of As^3+^ measurements and SenALiB-676n was found to be the most efficient nanosensor created. SenALiB-676n was successfully expressed in the living cells and can monitor the real time flux rates of As^3+^ dynamics in each cell type. The cells are viable upon giving the As^3+^ treatment for 4 hrs with that we can conclude that this is more than appropriate time to study the level of As^3+^ in the living cells and therefore SenALiB can be expressed to study the flux of As^3+^ at any time. This FRET-based sensor is a simple and reliable approach that provides high performance measurements of changes in the As^3+^ concentration throughout the cells non-invasively and with high resolution both spatially and temporally. The beauty of this sensing tool is that it is genetically encoded and uses fluorescence sensing assay technology to monitor and measure the rapid changes in the flux rates of arsenic (As^3+^) at real time and quickly reports them as fluorescence signals from living cells both at *in vitro* and *in vivo* level without requiring disruption and/or degradation of the biological samples. The best thing is that the toxicity level of arsenic can be measured in any type of cells.

## Methods

### Construction of arsenic nanosensor

ArsR belongs to the metal-responsive transcriptional regulator family of ArsR/SmtB and was exploited as the recognition element for designing the ratiometric arsenic sensor. The arsR gene (sequence derived from Kyoto Encyclopedia of Genes and Genomes database) was amplified by polymerase chain reaction (pcr) from the genomic DNA of *E*. *coli* DH10β using a set of primers. The forward primer had the sequence 5ʹ-CGGGGTACCATGTCATTTCTGTTACCCATCC-3ʹ that introduces a restriction site *Kpn*I at the start codon of the arsR gene. The reverse primer with *KpnI* site was prepared after removing three STOP codons from the C-terminus of the arsR gene and consisted of the sequence 5ʹ-CGGGGTACCAATGTTCTTACTGTCCCCGGAA-3ʹ. *Kpn*I restriction site is underlined. ECFP and Venus were also pcr amplified and all the three amplicons were cloned sequentially into a bacterial expression vector pRSET-B giving rise to a nanosensor construct pRSET-B_ECFP_ArsR_Venus. Traditional strategy was adopted to clone fluorophores ECFP and Venus in pRSET-B vector giving rise to pRSET-B_ECFP and pRSET-B_Venus that would act as controls in the experiments. Also, a negative control pRSET-B_ECFP_Venus was developed consists of the two fluorophores without metal binding domain. Gateway LR Clonase II enzyme was used following instructions from the manufacturer for transferring SenALiB-676n into the yeast destination vector pYES-DEST52 to create an expression clone. BY4742 strain of *S*. *cerevisiae* was selected as the eukaryotic host. Cells were grown in liquid YEPD medium with continuous stirring and adequate aeration at 30 °C. For mammalian expression, SenALiB-676n was exercised from the expression vector pRSET-B and inserted into the pcDNA3.1(−) at *Bam*HI/*Hind*III sites.

### Protein expression and purification

pRSET-B_ECFP_ArsR_Venus was transferred to *E*. *coli* BL21 (codon plus) by heat shock method for bacterial cell expression of the nanosensor protein. The cells were grown in luria bertini (LB) broth at 37 °C containing antibiotics (ampicillin 100 µg/ml; chloramphenicol 10 µg/ml) until OD_600_ of 0.6 is reached. The expression was carried out using 0.5 mM IPTG (Himedia) and the cells were grown for the next 22 h at 20 °C under dark conditions. Cell harvesting was done in a centrifuge (4500 rpm for 20 min) and the pellet was resuspended in Tris–Cl buffer (20 mM, pH 8.0). Bacterial cells were lysed using a sonicator (Labsonics, USA) and the cell debris was removed by applying a centrifugal force of 4500 rpm for 20 min. Furthermore, the supernatant was loaded on to the Ni-NTA (Qiagen, Germany) resin, mixed well and stored at 4 °C for 1 hr allowing binding of the recombinant His-tagged protein. The affinity column was then filled with the mixture. Once the beads have settled in the column, it was washed with a buffer containing 20 mM Tris–Cl and 10 mM imidazole (pH 8.0). High concentration of imidazole (250 mM) was used in a buffer containing 20 mM Tris–Cl, pH 8.0 to elute the bound protein from the affinity column. The purified sensor protein was stored at 4 °C overnight for proper folding to its native conformation^49^ and sensor protein purity was checked by 10% SDS-PAGE.

### Spectral profile of SenALiB and stability

Fluorescence emission spectra were obtained using 420/20 nm excitation filter for ECFP and recording the emission intensities of the donor and acceptor at 485 nm and 540 nm respectively in the wavelength range 460–600 nm. Stability of SenALiB was analysed in various buffer systems with altered pH. Stability experiments were adapted from the previously published protocols^14^. Ratiometric changes in the emission intensities of the two fluorophores were recorded after diluting the sensor protein in 20 mM PBS, MOPS and Tris-Cl buffers of altered pH using a monochromator microplate reader. To test for the stability, FRET signal was determined with 20 mM MOPS buffer in respect to the altered pH range in absence and after adding 10 µM As^3+^.

### Fluorescence measurement

Intrinsic fluorescence measurements of the purified proteins expressed from constructs pRSET-B_ECFP, pRSET-B_Venus and pRSET-B_ECFP_Venus were performed to determine whether the increased FRET was a result of conformational changes induced in the binding domain ArsR or due to the effect of As^3+^ on individual fluorophore (ECFP/Venus). The sensor protein samples in 20 mM MOPS buffer were excited at λ_280_ & λ_420_ nm and the continuous profile of the emission intensities of ECFP and Venus was recorded at 310–400 nm and 460–500 nm wavelength range respectively on a monochromator reader with 96-well microtitre plate. Corresponding blank readings are subtracted each time to obtain the final fluorescence spectra. All readings were taken in triplicates. Fluorescence intensity was recorded in the presence of increasing concentration of As^3+^ in the concentration range 1–70 µM.

### *In vitro* characterization and ligand binding assay

Initial characterization of the protein was carried out by investigating the detection specificity of the nanosensor. FRET ratio was recorded with selected metal ions like arsenite, arsenate, cadmium, zinc, iron and mercury at different concentrations.

To perform the competitive experiments, FRET acceptor-to-donor fluorescence ratio was determined by adding the interferants such as NaCl (2 mM), KCl (150 mM), CaCl_2_ (10 µM), MgCl_2_ (10 mM), 20% (w/v) Ficoll 70, 20% (w/v) Dextran 70, 20% (v/v) PEG 400, ATP (5 mM) and glutathione (10 mM) to the sensor protein in the presence of 10 µM As^3+^. FRET ratio was recorded after adding 20 µl of these species to 180 µl of the diluted protein sample.

The *K*_d_ of SenALiB was measured after mixing the sensor protein (diluted in 20 mM MOPS buffer, pH 8.0) with different concentrations of As^3+^. To determine the affinity constant, ligand saturation curve was fitted in the binding isotherm equation^50^: S = (r − r_apo_)/(r_sat_ − r_apo_) = [As^3+^]/(*K*_d_ + [As^3+^]), where S is saturation of the binding site; r is ratio; r_apo_ is ratio in the absence of arsenite; r_sat_ is ratio at saturation with arsenite and [As^3+^] is arsenite concentration. All the readings were taken in triplicates in a 96-well microtitre plate.

### Mutagenesis for creating sensor variants

Affinity mutants were developed to increase the sensor’s detection range for As^3+^. Mutations were introduced using QuikChange II site-directed mutagenesis kit (Agilent, USA) in the sensor protein to change its affinity. The sensor variants were generated by substituting the residues glutamic acid, alanine and arginine/glutamic acid at positions 27, 81 and 55/56 of ArsR protein by proline, tryptophan and isoleucine/glutamine respectively. The mutants E27P, A81W and R55I-E56Q of SenALiB were purified as stated above. Variant SenALiB-676n was used further for carrying out experimental assays at *in vivo* level.

### *In vivo* measurement of As^3+^ by SenALiB-676n in bacterial cells

SenALiB-676n was transformed in *E*. *coli* BL21-codon plus for *in vivo* analysis of the nanosensor in bacterial cells. The bacterial cells were grown in the LB medium, induced by 0.5 mM IPTG for 22 h in the dark at 20 °C after the cells were in their log phase to allow the expression of nanosensor protein. The cells were harvested, and the pellet was resuspended in 20 mM MOPS buffer (pH 7.0). 10 µM As^3+^ was added to 180 µl of the bacterial cell suspension and the fluorescence emission intensity ratio was recorded for 80 min at regular interval of 5 min. Titration assay was carried out in triplicates. As negative control, ECFP-Venus construct was cloned in pRSET-B. Control experiment was carried out simultaneously using ECFP-Venus system and the FRET ratio was monitored for 80 min after addition of 10 µM As^3+^. Images of the bacterial cells were acquired using confocal microscope (Leica DMRE) equipped with TCS-SPE confocal head and LAS-AF software.

### Monitoring of arsenic uptake in yeast cells

As^3+^ uptake mechanism in yeast was studied by transforming BY4742 strain *S*. *cerevisiae* with of SenALiB-676n. The cells were grown for 3–5 days in synthetic defined (SD)-growth medium with 2% sucrose and 3% galactose as carbon source and inducer respectively for the expression of nanosensor^14^. *S*. *cerevisiae* was chosen for *in vivo* measurement of As^3+^ flux dynamics and ratiometric images of the yeast cells expressing the sensor were also taken by fixing the cells on a poly L-lysine coated cover slide. The cells were incubated with 10 µM As^3+^ and FRET ratio change was recorded for 10 min using LAS-AF software (excitation 420/20 nm; emission filters 485/20 nm and 540/20 nm). As negative control, ECFP-Venus construct was cloned in yeast expression vector pYEST-DEST52. 10 µM As^3+^ was added to the ECFP-Venus system and dual emission intensity ratio changes were obtained for the defined period. Video was recorded to visualize the ratiometric changes due to the flux of As^3+^ in a single cell (Supplementary Video 1).

### Intracellular detection of arsenic uptake in HEK cells

For mammalian cell expression, HEK-293T cells were cultured in Dulbecco’s Modified Eagle’s Medium (DMEM, Sigma, USA) at 37 °C. The cells were maintained in a CO_2_ humidifier chamber with an antibiotic (ampicillin 50 μg/ml) and10% fetal calf serum. HEK-293T cells were grown in culture plates with 6-well and transiently transfected with SenALiB-676n by calcium phosphate method. Foe next two days, the cells were cultured for expression of the nanosensor. The cells were washed with PBS buffer (pH 7.2) and fluorescence measurements were performed after incubating the cells with 10 µM As^3+^ using the confocal microscope. As negative control, ECFP-Venus construct was cloned in mammalian cell expression vector pcDNA3.1 (−). A negative control was established wherein 10 µM As^3+^ added to the ECFP-Venus system and ratio changes were recorded for the set period. Region of interest (ROI) was picked and background subtraction was done to prevent artifacts in the FRET signal. Ratiometric imaging of the cells were performed and 10 min video was recorded on a confocal microscope with 1.53 N.A., 63x oil immersion objective and cooled charge coupled camera^45^ to visualize the changes in the dual emission intensity ratio in a single cell (Supplementary Video 2).

### Cell culture and toxicity tests

HEK-293T cell line was maintained in complete DMEM medium supplemented with 10% heat inactivated fetal bovine serum (FBS), 1% streptomycin, amphotericin B and penicillin solution in a humidified 5% CO_2_ incubator, 37 °C. Cell cultures were routinely cultured and trypsinized not more than 30 passages. To assess the cytotoxic potential of As^3+^, MTT assay was carried out by following previously published protocols^51–53^. Briefly, HEK-293 cells were plated at a seeding density of 9000–10000 cells/well in a 96-well cell culture plate and grown overnight. Cells were treated with different concentrations of As^3+^ (0–100 µM) for 4 hr and 16 hr. Succeeding the incubation time, mixture of culture medium and arsenic were removed and each well of the cells were washed with PBS pH 7.4. Mixture of 100 µl serum free DMEM and 25 µl MTT solution (from 5 mg/ml stock) were added to each well and incubated for 4–5 hr at 37 °C in the CO_2_ incubator. After incubation, supernatant was removed and purple formazan crystals were dissolved in 150 µl of dimethyl sulfoxide (DMSO). The absorbance of final reaction products was read out at 570 nm using a multiplate ELSIA reader (BioRad). The percentage cell viability was estimated and plotted as a function of concentration of As^3+^. In case of direct imaging, cells were plated in 12 well cell culture plate and treated with 10 µM and 100 μM As^3+^, control cells were treated with media only. Cell images were taken using phase contrast inverted microscope and morphological changes were studied.

## Supplementary information

Supplementary Fig. S1,S2,S3,S4,S5,S6,S7

Supplementary video 1

Supplementary video 2
