# Supplementary material for: Engineering genetically encoded FRET-based nanosensors for real time display of arsenic (As3+) dynamics in living cells
Source: Sci Rep. 2019 Aug 2;9:11240. doi: 10.1038/s41598-019-47682-8 (PMC6677752; doi:10.1038/s41598-019-47682-8)

**SUPPLEMENTARY INFORMATION**

**Engineering genetically encoded FRET-based nanosensors for real time display of arsenic (As^3+^) dynamics in living cells.**

Neha Soleja^1^, Ovais Manzoor^1^, Parvez Khan^2^ and Mohd. Mohsin^1^*

^1^Department of Biosciences, Jamia Millia Islamia, New Delhi-110025, India

^2^Centre for Interdisciplinary Research in Basic Science, Jamia Millia Islamia, New Delhi-110025, India

*Corresponding author: M. Mohsin ([mmohsin1@jmi.ac.in](mailto:mmohsin1@jmi.ac.in)).

Metabolic Engineering Laboratory, Department of Biosciences, Jamia Millia Islamia, New Delhi-110025, India

Phone no: +91-(11)26981717

Fax: +91(11)2698 0229

**Supplementary Fig. S1.** Linear arrangement of the sequences in the nanosensor construct ECFP-arsR-venus.


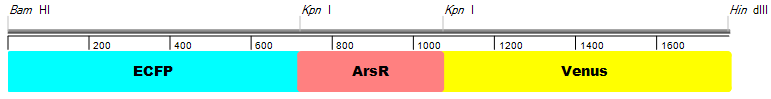


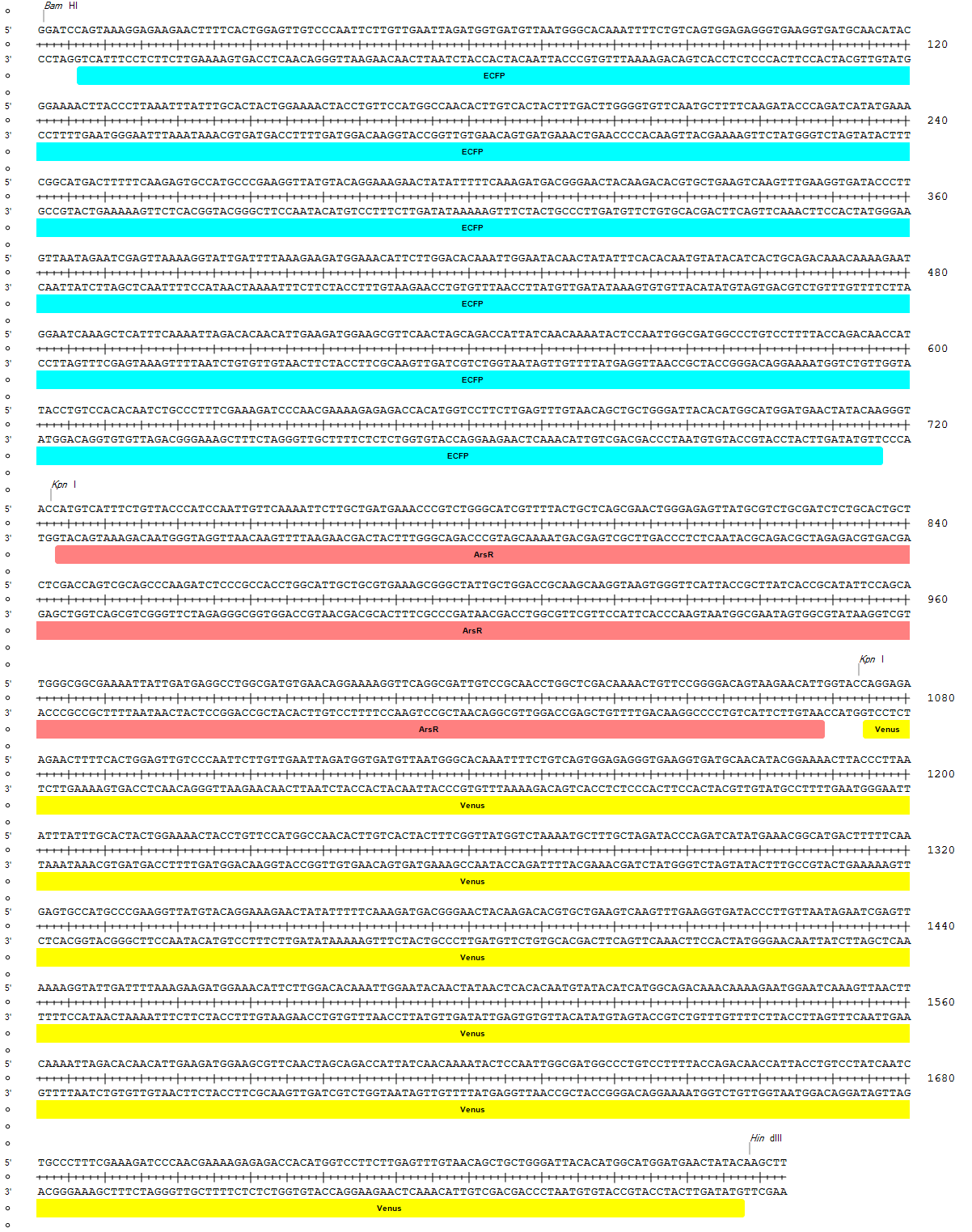


**Supplementary Fig. S2.** The nanosensor construct maps in different expression vectors.

**Supplementary Fig. S3.** BLAST result of arsR sequence. Query represents original sequence of the gene and subject represents the sequencing result.


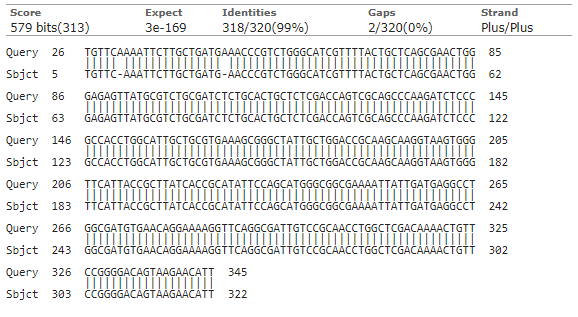


**Supplementary Fig. S4.** Effect of As^3+^ on the fluorophores. Fluorescence emission spectra of (**a**) ECFP (**b**) Venus and (**c**) ECFP-Venus proteins by exciting at λ_280_ nm.

**a**

**E**LVETICA


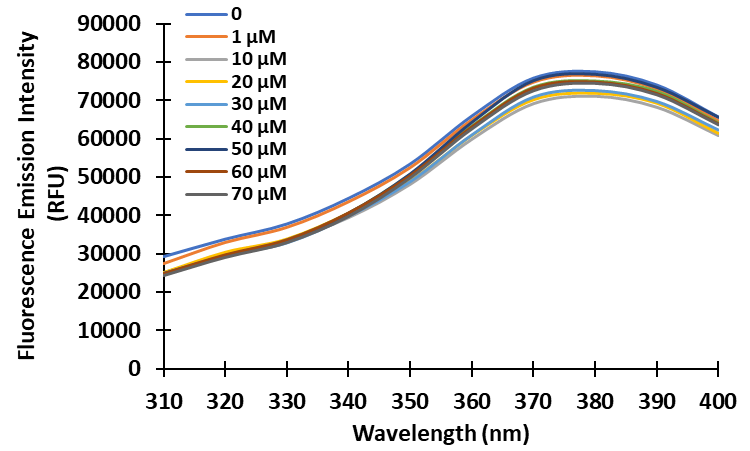


**b**

**E**LVETICA


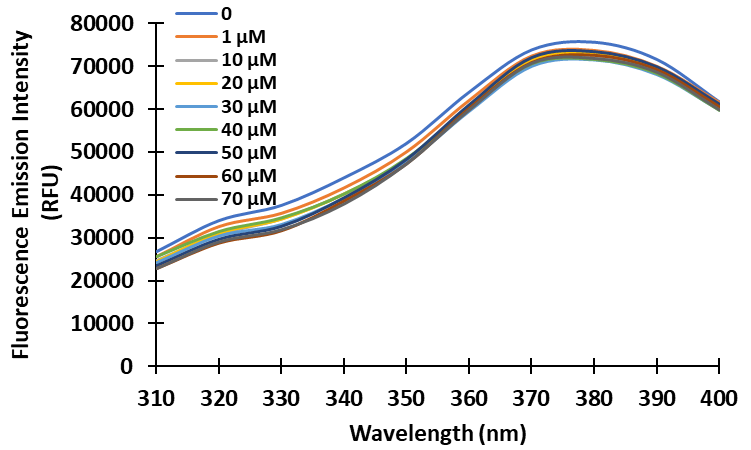


**c**

**E**LVETICA


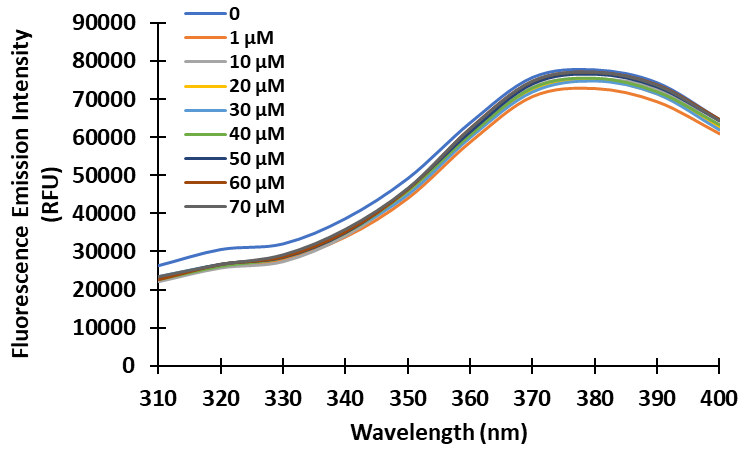


**Supplementary Fig. S5.** Effect of As^3+^ on the fluorophores. Fluorescence emission spectra of (**a**) ECFP (**b**) Venus and (**c**) ECFP-Venus proteins with the excitation at λ_420_ nm.

**a**

**E**LVETICA


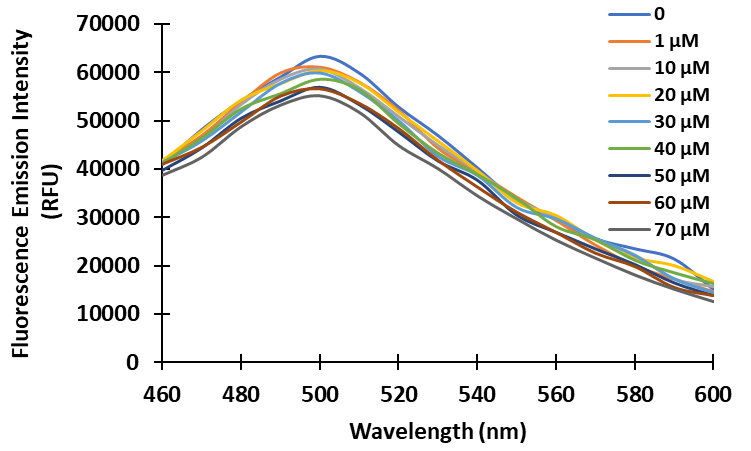


**b**

**E**LVETICA


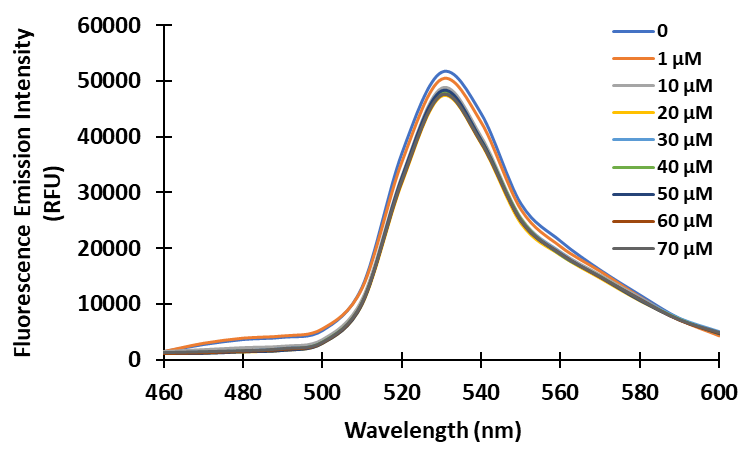


**c**

**E**LVETICA


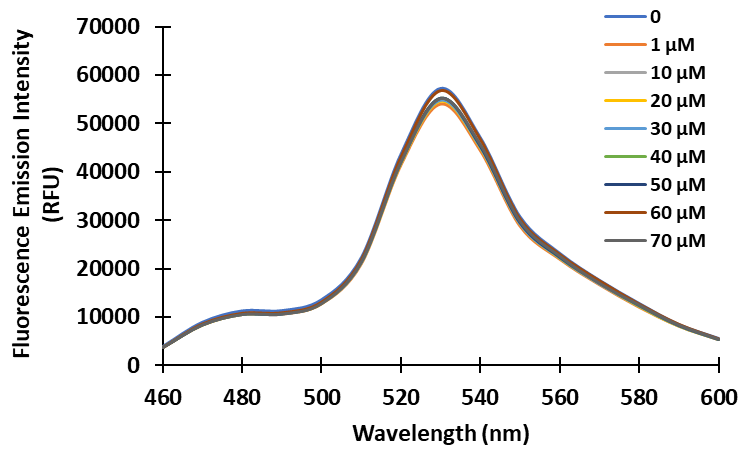


**Supplementary Fig. S6.** Dendogram showing (**a**) Mutation at position 27 convert glutamic acid to proline (GAA to CCA). (**b**) Mutation at position 81 convert alanine to tryptophan (GCG to TGG). (**c**) Mutation at position 55 and 56 convert arginine to isoleucine (CGT to ATT) and glutamic acid to glutamine (GAA to CAA) respectively.

**a**

**E**LVETICA

**
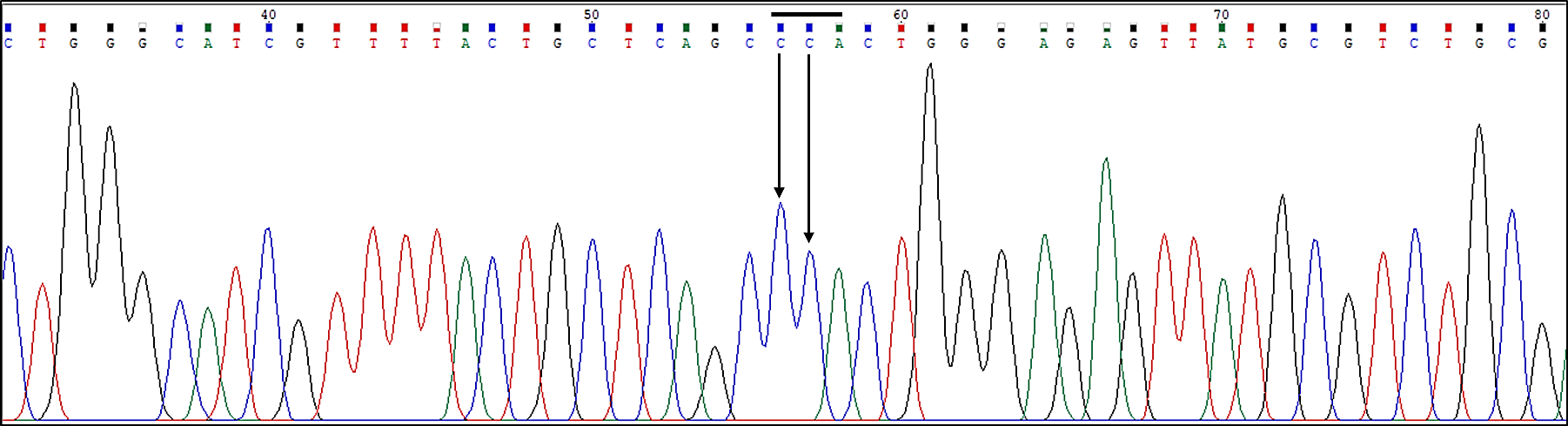
**

**b**

**E**LVETICA

**
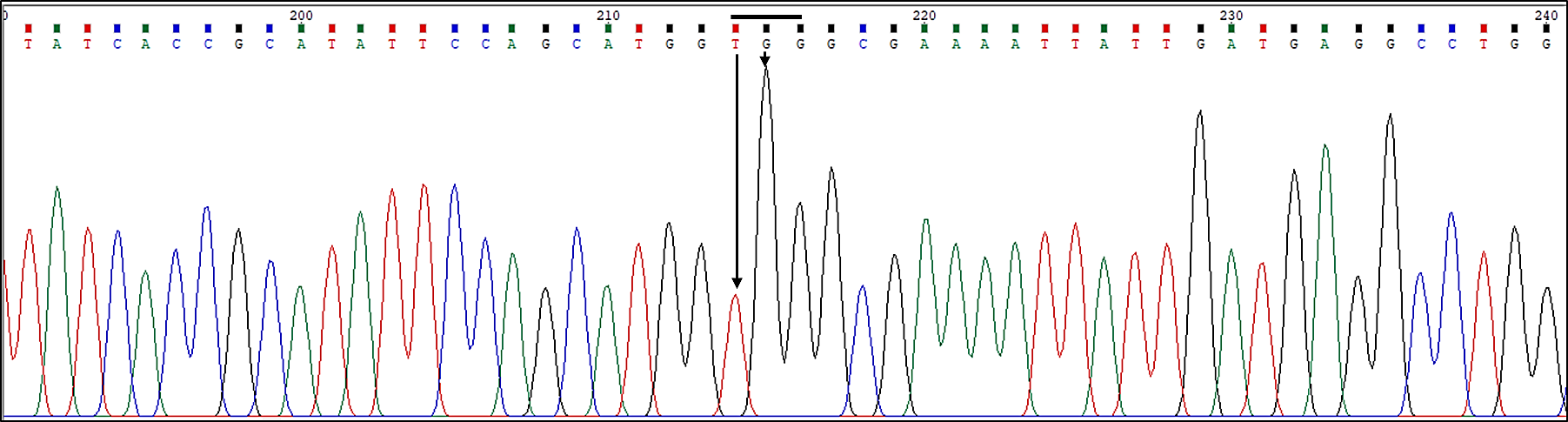
**

**c**

**E**LVETICA


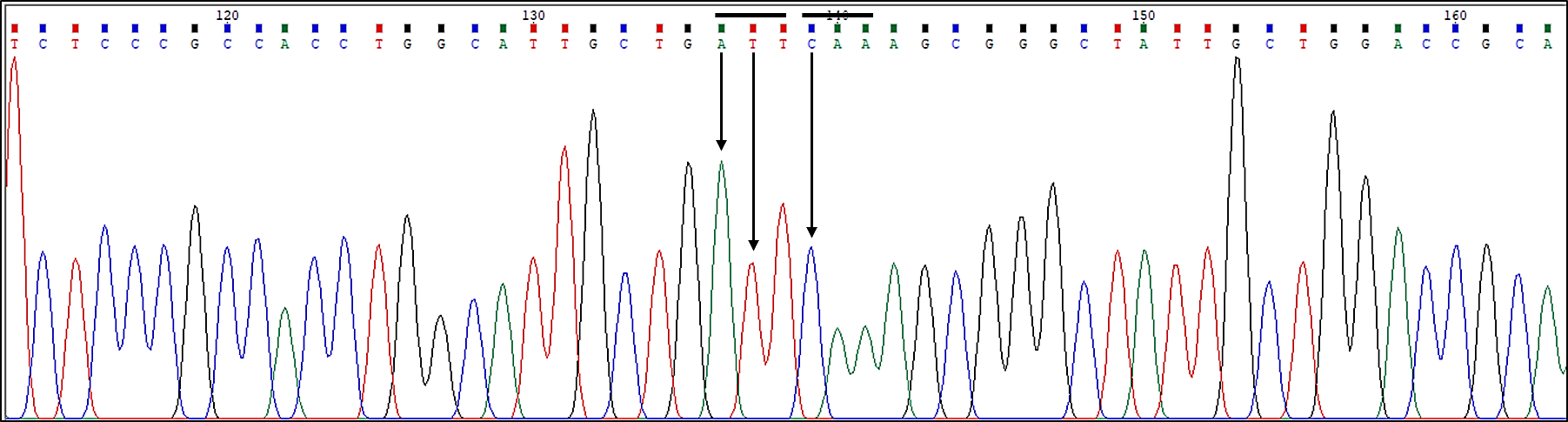


**Supplementary Fig. S7.** Confocal imaging of the bacterial cells expressing the genetically encoded SenALiB-676n.


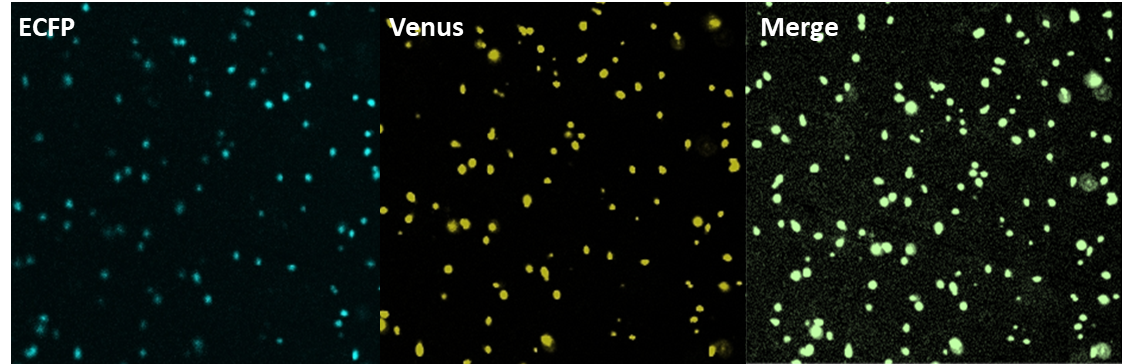

Supplement: Supplementary file 1 — Supplementary Fig. S1,S2,S3,S4,S5,S6,S7 [file 41598_2019_47682_MOESM1_ESM.doc]
